# Metabolic Characteristics of Porcine LA-MRSA CC398 and CC9 Isolates from Germany and China via Biolog Phenotype MicroArray^TM^

**DOI:** 10.3390/microorganisms10112116

**Published:** 2022-10-26

**Authors:** Henrike Krüger-Haker, Xing Ji, Alexander Bartel, Andrea T. Feßler, Dennis Hanke, Nansong Jiang, Karsten Tedin, Sven Maurischat, Yang Wang, Congming Wu, Stefan Schwarz

**Affiliations:** 1 Institute of Microbiology and Epizootics, Department of Veterinary Medicine, Freie Universität Berlin, 14163 Berlin, Germany; 2 Veterinary Centre for Resistance Research (TZR), Freie Universität Berlin, 14163 Berlin, Germany; 3 Jiangsu Key Laboratory for Food Quality and Safety, State Key Laboratory, Cultivation Base of Ministry of Science and Technology, Institute of Food Safety and Nutrition, Jiangsu Academy of Agricultural Sciences, Nanjing 210000, China; 4 Institute for Veterinary Epidemiology and Biostatistics, Department of Veterinary Medicine, Freie Universität Berlin, 14163 Berlin, Germany; 5 Key Laboratory of Animal Antimicrobial Resistance Surveillance, MARA, College of Veterinary Medicine, China Agricultural University, Beijing 100193, China; 6 Department Biological Safety, German Federal Institute for Risk Assessment (BfR), 10589 Berlin, Germany

**Keywords:** *Staphylococcus aureus*, metabolic properties, area under the curve (AUC), sparse partial least squares discriminant analysis (sPLS-DA), whole-genome sequencing (WGS)

## Abstract

Livestock-associated methicillin-resistant *Staphylococcus aureus* (LA-MRSA) is an important zoonotic pathogen, often multi-resistant to antimicrobial agents. Among swine, LA-MRSA of clonal complex (CC) 398 dominates in Europe, Australia and the Americas, while LA-MRSA-CC9 is the main epidemic lineage in Asia. Here, we comparatively investigated the metabolic properties of rare and widespread porcine LA-MRSA isolates from Germany and China using Biolog Phenotype MicroArray technology to evaluate if metabolic variations could have played a role in the development of two different epidemic LA-MRSA clones in swine. Overall, we were able to characterize the isolates’ metabolic profiles and show their tolerance to varying environmental conditions. Sparse partial least squares discriminant analysis (sPLS-DA) supported the detection of the most informative substrates and/or conditions that revealed metabolic differences between the LA-MRSA lineages. The Chinese LA-MRSA-CC9 isolates displayed unique characteristics, such as a consistently delayed onset of cellular respiration, and increased, reduced or absent usage of several nutrients. These possibly unfavorable metabolic properties might promote the ongoing gradual replacement of the current epidemic LA-MRSA-CC9 clone in China with the emerging LA-MRSA-CC398 lineage through livestock trade and occupational exposure. Due to the enhanced pathogenicity of the LA-MRSA-CC398 clone, the public health risk posed by LA-MRSA from swine might increase further.

## 1. Introduction

Livestock-associated methicillin-resistant *Staphylococcus aureus* (LA-MRSA) has shown an increase in food-producing animals since the mid-2000s [1]. This MRSA variant was first recognized in pigs [2,3] and later in other livestock, such as cattle or poultry [1]. LA-MRSA are considered a matter of public health concern since such isolates are often multi-resistant to antimicrobial agents and can easily cross species barriers [1,4]. Several reports on LA-MRSA transmission from animals to humans point out the increased risk of also becoming MRSA carriers for persons in close contact with LA-MRSA-positive animals [1,4,5,6,7]. Healthy, colonized pigs represent one of the main reservoirs for LA-MRSA [5]. Such isolates only rarely cause diseases in swine ranging from skin infections to pneumonia or septicemia [8]. However, LA-MRSA can also cause a broad spectrum of mild to severe infections in humans although outbreaks have been reported only sporadically [4,5,6]. 

Detailed molecular analyses revealed that LA-MRSA colonizing pigs in Europe, Australia as well as North and South America belong predominantly to the clonal complex (CC) 398 [8,9,10]. In contrast, CC9 was identified as the dominant pig-associated clonal lineage in most Asian countries, particularly in China [11,12,13]. In line with this, LA-MRSA CC9 isolates in European countries and LA-MRSA CC398 isolates in China were only rarely isolated from swine [9,14]. However, reports of LA-MRSA CC398 isolates from China have recently increased [15,16,17]. Previous studies on LA-MRSA CC398 and CC9 mainly investigated host adaptation and pathogenicity of the isolates or focused on genetic analyses and epidemiological relationships [8,9,10,16,17,18,19,20,21,22]. Although prior studies have helped to clarify the impact of genomic characteristics, fitness and virulence of LA-MRSA CC398 and CC9 isolated from China and Germany on the development of two pig-associated epidemiologically successful LA-MRSA clones in different regions in a comparative study [23], some questions remain unanswered.

Biolog Phenotype MicroArray^TM^ (PM) technology for microbial cells offers a high-throughput method for the extensive analysis of cellular phenotypes [24,25]. Responses of a microbial community or an individual isolate to numerous classes of chemical compounds can be measured using a colorimetric redox reaction that mirrors the extent of cellular respiration. By investigation of susceptibility to antimicrobial agents and metabolic pathways along with ionic, osmotic as well as pH effects, the metabolic and chemical sensitivity properties of microbial cells can be determined. The Biolog PM system has been widely used in a variety of fields to gain an overview of a microbial community’s metabolic profile or to study specific gene functions by detecting phenotypic changes associated with gene knockouts [25,26]. The *S. aureus* small-colony variants (SCVs), for example, are increasingly found in antibiotic-refractory as well as recurrent infections and are known to exhibit several phenotypic changes [27]. Von Eiff et al. conducted Biolog PMs of SCV mutants to define metabolic variations between SCVs and wild-type *S. aureus* more precisely, revealing defects in ATP generation via electron transport due to defects in carbon metabolism and proving growth of SCV mutants utilizing carbon sources that provide ATP independently from electron transport [27]. Another study focused on phenotypic characterization of *S. aureus sarR* mutant isolates [28]. The staphylococcal specific *sar* family genes control the expression of factors associated with virulence, antimicrobial resistance and survival under adverse conditions [28]. Biolog PM analyses allowed identification of altered utilization of multiple substrates, which suggested that *sarR* might play a role in controlling cell wall or membrane related functions [28]. Furthermore, Marchi et al. performed Biolog PM studies to investigate antimicrobial resistance and susceptibility phenotypes associated with isolates harboring mutations up-regulating different efflux pumps [29]. They identified compounds to which the mutant isolates showed enhanced susceptibility, suggesting potential opportunities to overcome the antimicrobial resistance problem [29]. Most recently, Vaillant et al. investigated the use of the Biolog PM system for antimicrobial susceptibility testing in comparison to broth microdilution using clinical *S*. *aureus* isolates [30]. Despite these prior applications regarding the connections between virulence, antimicrobial resistance and metabolic traits in *S. aureus*, to the best of the authors’ knowledge, Biolog PM analyses have not been used to characterize LA-MRSA isolates.

In this study, we examined a representative collection of whole-genome sequenced LA-MRSA CC398 and CC9 isolates of swine from Germany and China for possible differences in their metabolic properties applying Biolog PM technology to evaluate whether metabolic variations could have played a role in the development of two different epidemic LA-MRSA clones in swine.

## 2. Materials and Methods

### 2.1. Selection of Representative Bacterial Isolates

In total, 20 whole-genome sequenced porcine LA-MRSA isolates were chosen as representative test collection, including five dominant MRSA-CC398 from Germany (GER-MRSA-CC398) and five dominant MRSA-CC9 from China (CHN-MRSA-CC9) as well as five rare MRSA-CC9 from Germany (GER-MRSA-CC9) and five rare MRSA-CC398 from China (CHN-MRSA-CC398). Their characteristics are displayed in Table 1. All isolates were obtained from swine during the period between 2004 and 2017 in various regions of Germany and China [23]. As only the five CHN-MRSA-CC398 isolates investigated were available to us during the study period, the other three groups were set up to be comparable in isolate number. Here, independent isolates were selected based on preferably diverse pheno- and genotypic antimicrobial resistance profiles. The antimicrobial susceptibility testing data were obtained in a previous study [23]. The 15 antimicrobial agents tested included cefoxitin, tetracycline, minocycline, tigecycline, erythromycin, clindamycin, virginiamycin M1, gentamicin, ciprofloxacin, florfenicol, tiamulin, trimethoprim, linezolid, vancomycin and fusidic acid. Furthermore, preliminary Biolog PM experiments confirmed, in general, a uniform metabolic behavior of the isolates within each of the four groups. Therefore, the selected isolates were considered as representative for the respective LA-MRSA category.

### 2.2. Whole-Genome Sequencing, Assembly and Annotation

Genomic DNA was extracted using a HiPure Bacterial DNA Kit (Magen, Guangzhou, China). The libraries were prepared using the KAPA Hyper Prep Kit (Kapa Biosystems, Boston, MA, USA) and sequencing was performed on the Illumina HiSeq X-Ten System (Annoroad Genomics Co., Beijing, China). For each isolate, 300-bp paired-end reads with a minimum of 250-fold coverage were obtained. SPAdes (version 3.12.0) [35] was used for assembly of the DNA sequence reads, which were then run through an automatic annotation pipeline via RAST [36]. The whole-genome sequences of the 20 MRSA isolates included in this study have been deposited at GenBank under the accession numbers JAMYYK000000000, JAMYYJ000000000, JAMYYI000000000, JAMYYH000000000, JAMYYG000000000, JAMYYF000000000, JAMYYE000000000, JAMYYD000000000, JAMYYC000000000, JAMYYB000000000, JAMYYA000000000, JAMYXZ000000000, JAMYXY000000000, JAMYXX000000000, JAMYXW000000000, JAMYXV000000000, JAMYXU000000000, JAMYXT000000000, JAMYXS000000000, and JAMYXR000000000 (see also the Data Availability section).

### 2.3. Molecular and Phylogenetic Analyses

Known genes conferring antimicrobial resistance were located by directly mapping the DNA sequence reads against the ResFinder database [37] applying a procedure implemented in the SRST2 tool [38]. The contigs generated with SPAdes were also checked for the respective genes using AMRFinderPlus [39]. Results obtained from both databases were compared and verified using Geneious v11.1.4 (Biomatters Ltd., Auckland, New Zealand). Moreover, chromosomal point mutations associated with antimicrobial resistance were located applying PointFinder [40], which is implemented in the ResFinder tool [37] of the Center for Genomic Epidemiology (CGE). Multilocus sequence typing (MLST), *spa* typing and SCC*mec* typing were carried out using the MLST 2.0 [41], *spa*Typer 1.0 [42] and SCC*mec*Finder 1.2 [43] CGE online analysis tools. The *dru* types were determined from the dru-typing.org database [44] by using the blast function in Geneious v11.1.4. Ridom SeqSphere+ (version 7.5.5) [45] was used for phylogenetic analysis via the *S. aureus* core genome MLST (cgMLST) approach [46]. In order to illustrate the clonal relationship between the representative isolates, a minimum spanning tree was built based on a distance matrix of the core genome allelic profiles including 1749 of 1861 possible target genes by removing 112 columns with missing values from the comparison table.

### 2.4. Biolog PM Assay Performance and Data Evaluation

Materials, chemicals, instrumentation and consumables for the Biolog PM assays were obtained from Biolog Inc., Hayward, CA, USA. The Biolog PM assays were conducted using the 96-well PM1 (carbon utilization), PM2A (carbon utilization), PM9 (osmotic and ionic effects), and PM10 (pH effects) microplates, which represent different metabolic capabilities and/or growth conditions [24]. The experiments were carried out at least in independent duplicates according to the manufacturer’s PM procedures for Gram-positive bacteria with slight modifications of the incubation conditions. Bacteria from the frozen stock cultures were grown overnight at 37 °C on BUG + B (Biolog Universal Growth Medium + 5% sheep blood) agar plates. After subculturing for a second time, bacterial cells were removed from the BUG + B plate and transferred into sterile IF-0a GN/GP Base fluid. Uniform cell suspensions with a final cell density of 81% T (transmittance) were combined with the respective inoculating fluid depending on the microplate to be used, according to the manufacturer’s recommendations. The recipes varied slightly in the composition of IF-0a GN/GP Base and IF-10b GN/GP Base fluid, the redox dye tetrazolium violet (Dye H), and different additive solutions. The Biolog PM microplates were inoculated with the final cell suspension (100 µL/well) and incubated at 37 °C in the OmniLog incubator. The OmniLog PM system (OmniLog Data Collection software version 2.3.01) recorded the colorimetric data every 15 min for 48 h. In order to detect abiotic “false-positive” reactions, an additional set of control Biolog PM microplates (PM1, PM2A, PM9, PM10) was incubated using the same assay protocol described above but without the bacterial cells. 

The final kinetic Biolog PM data were processed and evaluated using OmniLog Data File Converter, OmniLog FM (version 1.20.02), OmniLog PM (version 1.20.02), and Biolog Data Analysis (version 1.7.1.51) software. Kinetic graphs obtained from the colorimetric reaction in each well were designated as “respiration curves” as they display the bacterial cells’ respiration over time. Respiration curves obtained after 24 and 48 h of incubation were compared visually between the four investigated groups. Moreover, metabolic substrate utilization was quantified via the parameters: area under the curve (AUC), lag time (LT), maximum height (MH), plateau time (PT) and slope of the respiration curves. Two statistical approaches were applied to obtain possible selections of the most informative substrates and/or conditions tested. Firstly, sparse partial least squares discriminant analysis (sPLS-DA) [47] was employed considering two independent AUC measurements obtained after 24 h of incubation for each isolate and all wells/conditions investigated. sPLS-DA provides variable selection (Lasso) for datasets with a large number of highly correlated variables [47]. The optimal number of selected variables was determined using 10-fold cross validation. This approach revealed that four components containing 30 of the 379 substrates and/or conditions provided optimal discriminatory power to differentiate the four MRSA groups. Components combine similarly behaving variables into groups (instrumental variables). This aids in selecting variables according to recurring patterns and reduces the risk of selecting variables based on randomly occurring differences in ‘omics datasets. Based on the selected variables, a heatmap was generated consisting of one row per isolate and one column per substrate. The colored tiles represent the average z-score for each isolate and substrate combination. A hierarchically clustered tree was generated using the Euclidian distances between isolate (rows) and substrate (columns) z-scores. Secondly, sPLS-DA [47] analysis was carried out for each of the parameters LT, MH, PT and slope considering independent duplicate measurements obtained after 24 h of incubation for each isolate and all wells investigated. Parameters were ranked according to variable importance in each of the analysis for every parameter. Here, 40 of the 379 substrates and/or conditions tested were selected based on the lowest combined rank between all four analyses. This combined sPLS-DA variable selection was performed since AUC values can differ due to varying reasons, such as clearly different curves or just minor changes in the maximum height. This analysis favors variables with differences in respiration curves in more than one curve parameter. All of the statistical analyses were performed using the R package mixOmics (version 6.18.1) [48] and R version 4.1.3 (R Foundation for Statistical Computing, Vienna, Austria).

## 3. Results and Discussion

### 3.1. Molecular and Phylogenetic Analyses

Molecular typing results and antimicrobial resistance data are displayed in Table 1. All porcine LA-MRSA from Germany and China displayed *spa* and SCC*mec* types that are typically associated with CC9 and CC398, respectively [8,21,49]. An exception is *spa* type t571, which was found in isolate SHP1 and has so far rarely been reported in MRSA sequence type (ST) 398 isolates [50,51]. However, a recent study has shown that swine-associated MRSA ST398 circulating in a slaughterhouse in China were predominantly associated with *spa* type t571 [17]. The methicillin-susceptible *S. aureus* (MSSA) t571 clonal lineage also predominated in swine, human, and environment-associated isolates [17]. Moreover, MSSA of ST398 and *spa* type t571 have been reported from human infections in Belgium [52], China [53], Colombia [54], France [55,56], Germany [51], the Netherlands [50], and the United States [57]. It is worth noting that several of the affected patients had no or only indirect contact with livestock prior to infection. Interestingly, *spa* type t571 harbors only one repeat (r24) less than type t034, which is commonly detected in MRSA-CC398.

The antimicrobial resistance data were used for selection of representative isolates for Biolog PM assays as described above. In general, antimicrobial resistance patterns of LA-MRSA from China were more complex than those of LA-MRSA from Germany (Table 1). All tested isolates harbored several antimicrobial resistance genes, which differed in number and distribution among the isolates. The Chinese isolates displayed higher numbers of antimicrobial resistance genes, especially the CHN-MRSA-CC9. As MRSA, all isolates were resistant to beta-lactams and carried the *mecA* gene as well as at least one copy of the *blaZ* gene. In addition, all CHN-MRSA-CC9 and -CC398 were resistant to tetracycline, erythromycin, clindamycin and tiamulin. All GER-MRSA-CC398 furthermore revealed tetracycline resistance. However, all isolates were susceptible to linezolid and vancomycin and the German isolates additionally to florfenicol and tiamulin. The isolates’ resistance profiles mirror the selection pressure imposed by antimicrobial agents commonly used in the pig industry. Usage of tetracyclines, tiamulin, florfenicol, as well as macrolides, lincosamides and streptogramins (MLS) is associated with selection of multi-resistant isolates in China [58]. In Germany, MRSA isolates have adapted to selection pressure imposed by tetracyclines by acquiring the respective antimicrobial resistance genes [59].

**Figure 1:**
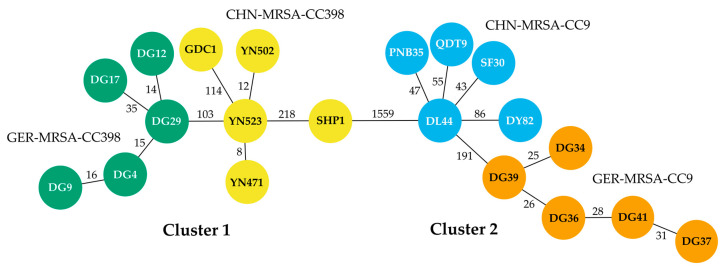
Minimum spanning tree displaying the phylogenetic relationship of 20 porcine MRSA isolates from Germany and China based on cgMLST analysis including 1749 alleles using the SeqSphere+ software. The circles represent distinct allelic profiles and the count of varying target genes between the different profiles is shown next to the connecting lines. The isolate IDs are given within the circles; origin and CC are indicated by color: GER-MRSA-CC398 in green, CHN-MRSA-CC398 in yellow, CHN-MRSA-CC9 in blue and GER-MRSA-CC9 in orange.

The cgMLST analysis revealed two different clusters, in accordance with the MLST and CC assignments (Table 1), and 20 varying allelic profiles for the 20 MRSA isolates (Figure 1, Table S1). Cluster 1 comprised the CC398 isolates and included five rather closely related allelic profiles each from Germany and China. The GER-MRSA-CC398 differed in 14 to 42 and the CHN-MRSA-CC398 in 8 to 241 target genes (Table S1). The two most closely related German (DG29) and Chinese (YN523) CC398 isolates varied in 103 target genes. Cluster 2 represented the CC9 sequences, also assigned to five more closely related allelic profiles each from Germany and China. The GER-MRSA-CC9 varied in 25 to 41 and the CHN-MRSA-CC9 in 43 to 108 target genes (Table S1). The two most closely related German (DG39) and Chinese (DL44) MRSA-CC9 differed in 191 target genes. According to a previous study on the phylogenetic analysis of three MRSA outbreaks applying the SeqSphere+ cgMLST approach, *S. aureus* isolates displaying 0 to 8 allelic differences should be considered as related; isolates with 9 to 29 allelic differences as possibly related; and those with ≥30 allelic variations as unrelated [60]. Thus, two CHN-MRSA-CC398 seemed to be related, while four GER-MRSA-CC398, four GER-MRSA-CC9 and three CHN-MRSA-CC398 showed varying possible relationships among each other (Table S1). Moreover, the differences in 1559 of 1749 alleles included in the comparison reflected the considerably distant clonal relationship between Cluster 1 and 2. As the isolates SHP1 within CHN-MRSA-CC9, and relatively higher numbers of allelic differences between the groups, the division of isolates into four groups for the Biolog PM assay based on their CC and origin was in accordance with the phylogenetic analysis (Figure 1, Table S1).

### 3.2. Biolog PM Assay Analysis

If not further specified, all findings described in this section relate to an incubation period of 24 h. Some of the chemical compounds used in the Biolog PM microplates can directly reduce Dye H even when no bacterial cells are added resulting in the formation of purple color [24]. These wells will give abiotic “false-positive” reactions without bacteria present because the dye is chemically reduced. There is no final list of abiotic wells available because the dye reduction depends on the definitive test parameters. Therefore, a set of Biolog PM microplates was run without bacteria. Wells that showed abiotic reactions under the study’s experimental conditions are given in Table S2. Comparison of the obtained curves with the reduction kinetics resulting from the Biolog PM assays involving bacterial cells enabled assessment of the “false-positive” reactions. Biological reduction was gradual over time, whereas abiotic reduction occurred rapidly, more like a step function. In addition, biological exceeded chemical reduction in the abiotic wells. In the end, abiotic reactions did not affect further evaluation of the Biolog PM assay data as they were easy to distinguish from biological reduction due to bacterial respiration.

#### 3.2.1. General Metabolic Properties of Porcine LA-MRSA from Germany and China

Overall, the porcine MRSA isolates were able to metabolize a broad variety of macromolecules, including a wide spectrum of different carbon sources. These nutrients comprised sugars (D-galactose, D-trehalose, D-mannose, etc.), sugar alcohols (sorbitol, adonitol, maltitol, etc.) or (di-, tri-, hydroxy-) carboxylic acids (formic acid, succinic acid, citric acid, glycolic acid, etc.). Furthermore, the bacteria utilized the emulsifier tween, a wide range of amino acids, a number of other acids (a-keto-valeric acid, caproic acid, quinic acid, etc.) and nucleosides such as adenosine, thymidine or uridine. *S. aureus* requires endogenous and/or exogenous sources of biosynthetic precursors, e.g., carbohydrates, amino acids, nucleic acids, for assembly reactions generating DNA, RNA and proteins [61]. Moreover, in *S. aureus* the de novo synthesis of precursors requires 13 biosynthetic intermediates, which are provided by central metabolic pathways [61]. Central metabolism is both catabolic, i.e., energy-generating, and anabolic, i.e., energy-requiring, and involves glycolysis, gluconeogenesis, the pentose phosphate pathway and the tricarboxylic acid cycle in *S. aureus* [61]. In line with our findings, it is known that *S. aureus* is able to catabolize many different macromolecules, e.g., amino acids or nucleic acids, to produce the 13 intermediates, but typically prefers to use carbohydrates when available [61]. The process of carbohydrate translocation into the cytoplasm involves a group of different enzymes/transporters, where specificity for a certain sugar is provided by particular components. However, *S. aureus* has at least 15 enzyme II components encoded within its genome, which also highlights the pathogen’s versatility in carbohydrate utilization, consistent with our results [61]. In some cases, substrate metabolism was slightly delayed (α-keto-butyric acid, N-acetyl-neuraminic acid, 2-hydroxy benzoic acid, etc.), or was reduced compared to other substances (i-erythritol, citraconic acid, D-tartaric acid). Only a few carbon sources could not be metabolized at all by the MRSA isolates, including glyoxylic acid, capric acid and itaconic acid, which emphasizes the remarkable versatility in nutrient utilization.

*S. aureus* has also evolved a number of cellular stress responses allowing it to persist in different host species and to survive in varying environmental settings [62]. The bacterium’s high osmotolerance, for example, supports growth in high-osmolarity body sites, e.g., skin and mucosal surfaces, or in food with high salt concentrations [62]. Consistent with this, the porcine LA-MRSA from Germany and China showed pronounced metabolic activity in the presence of many osmolytes in various and even high concentrations. In several studies, the role of cytoplasmic accumulation of K^+^ and solutes as glutamine or proline in the adaptation to settings with increased osmolarity is discussed [62]. Here, all isolates were resilient to increasing concentrations of potassium chloride, sodium sulfate and ethylene glycol up to 6%, 5% and 20%, respectively. Their metabolism was also invariable to sodium phosphate and ammonium sulfate in all concentrations tested. Except for a minor delay, increasing concentrations of sodium chloride up to 10% had no substantial effect on bacterial metabolic activity. Similar to sodium chloride, rising concentrations of urea up to 7% resulted only in slightly delayed metabolic activity. However, individual substances in certain concentrations could significantly delay, reduce or even inhibit cellular respiration. The combination of sodium chloride 6% and dimethylsulphonylpropionate appeared to significantly decelerate the onset of metabolism more for some isolates and less for others. Moreover, increasing concentrations of sodium formate up to 6% reduced metabolic capacity and notably delayed bacterial metabolism. The presence of sodium lactate at concentrations of 3% to 12% also led to increasing inhibition of metabolic activity. Already at 4%, the metabolic activity of a few isolates was only recognizable during a 48 h incubation period. The isolates also showed reduced metabolism in the presence of sodium benzoate at a pH value of 5.2. The reduced metabolism was apparent at 20 mM, only a few isolates were able to maintain metabolism at 50 mM, and no activity was detected at 100 mM or 200 mM except for single isolates during a 48 h incubation period. In addition, rising concentrations of sodium nitrate had only a minor impact on the extent of metabolic activity, while those of sodium nitrite significantly reduced metabolism.

The extent of bacterial metabolism was also dependent on ambient pH conditions. Values between 5 and 10 did not pose significant difficulties, except for a slight decrease in metabolic activity at acidic values of 5 to 6. In contrast, no metabolic activity was detected at acidic pH values of 3.5 to 4.5. Provision of different nutrients such as amino acids and others at a pH of 4.5 did not affect this observation with the exception of urea. Here, some isolates were able to fully develop their activity, after a period of adaptation, which was mainly visible only over 48 h of incubation. Many studies investigated the effect of pH on the growth of *S. aureus*, for example to develop strategies to control *S. aureus* growth and enterotoxin production in food-manufacturing processes. Lanciotti et al. showed that among various bacterial species examined, *S. aureus* was characterized by the highest sensitivity to pH changes [63]. Considering an interactive effect of pH, water activity, temperature and ethanol concentrations on the pathogen’s growth, predicted critical pH values ranged between 5 and 7 [63], which matches the pH values allowing bacterial metabolism observed in our study. A pH sensitivity within the range of 5 to 7 was also shown for *S. aureus* by Iyer et al. emphasizing the advantages of keeping the skin pH within an acidic range for maintenance of a balanced skin microbiome, including a lower level of *S. aureus* compared with other commensals [64].

In contrast to a previous study [23], no differences were recognized between isolates belonging to CC398 and CC9 regarding the survival under acidic and hypertonic conditions.

#### 3.2.2. Statistical Evaluation of the Biolog PM Assay Data

##### 3.2.2.1. AUC sPLS-DA

**Figure 2:**
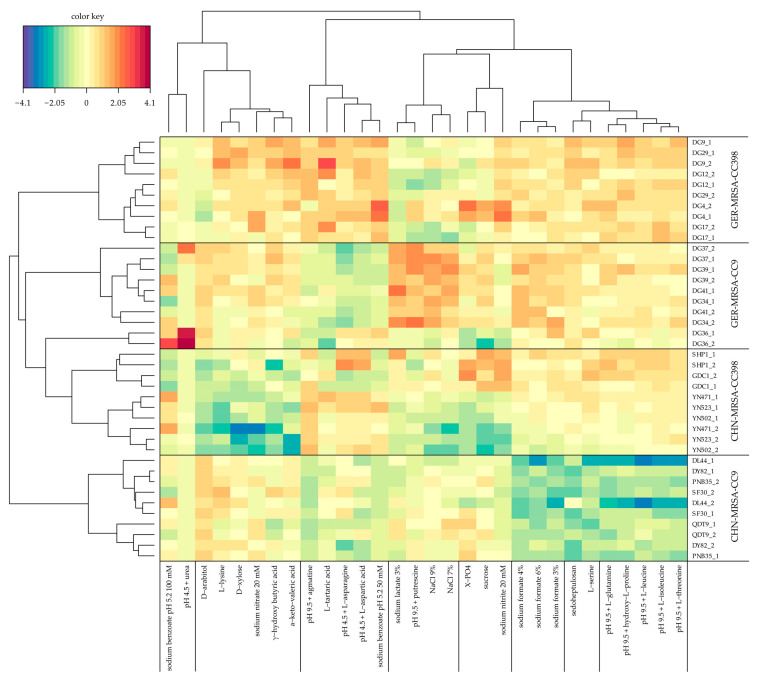
Heat map constructed via sPLS-DA using four components to differentiate porcine MRSA isolates from Germany and China based on CC and origin. Two independent area under the curve (AUC) values obtained after 24 h of incubation were considered for each of the isolates displayed on the right side. Based on the most remarkable differences selected nutrients are shown at the bottom. The color key mirrors the extent of metabolic substrate utilization (z-scaled) by the isolates, with red color displaying more successful and blue color less successful usage of a nutrient. The branching diagram provides classification of isolates and substrates into different groups.

The 30 sPLS-DA selected substances are displayed in Figure 2 and mainly included sugars, sodium salts and amino acids combined with varying pH conditions. The two independent AUC values considered in each case were reproducible. The branching diagram shows that differentiation of the isolates depending on their CC and origin into the four groups CHN-MRSA-CC9, CHN-MRSA-CC398, GER-MRSA-CC9 and GER-MRSA-CC398 was possible on the basis of the most remarkable differences in metabolic substrate utilization. Moreover, the selected substrates and/or conditions could be assigned to eight differing groups. Regarding the 30 most informative substrates, the heat map suggested that the groups GER-MRSA-CC398, GER-MRSA-CC9 and CHN-MRSA-CC398 harbored more similar metabolic profiles compared to the CHN-MRSA-CC9 (Figure 2). The Euclidian distance based on z-scores is for every Chinese CC9 isolate larger than that between the isolates of the other three groups. However, evaluation of respiration curves and mean AUC values revealed the following substrates as particularly interesting for the study’s research question: pH 4.5 + urea, D-arabitol, pH 9.5 + agmatine, sodium benzoate pH 5.2 50 mM, sodium formate 3%, sodium formate 4%, sodium formate 6%, sedoheptulosan, pH 9.5 + L-glutamine, pH 9.5 + hydroxy-L-proline, pH 9.5 + L-leucine, pH 9.5 + L-isoleucine, and pH 9.5 + L-threonine. The four groups also displayed crucial differences in the metabolism of several nutrients beyond the sPLS-DA selection, which are described in the following sections. Overall, the selection of substrates and/or conditions using this method allowed grouping of the isolates, but a more detailed insight into growth kinetics considering additional parameters to the AUC was required to evaluate metabolic differences between the four groups.

##### 3.2.2.2. Combined LT, MH, PT, and Slope sPLS-DA Variable Selection

The combined sPLS-DA variable selection also allowed a preselection of the most informative substrates and/or conditions tested. Similar to the AUC sPLS-DA selection, the 40 nutrients mainly included sugars, (amino) acids and sodium salts. In each case, the parameters LT, MH, PT and slope were reproducible and could be assessed in detail. Interestingly, the four isolate groups showed recurring patterns regarding the usage of many substances, which allowed classification of these metabolic differences as described in the following sections. However, for several substrates and/or conditions, the patterns were different across all parameters making classification impossible. Moreover, similar to the AUC sPLS-DA, relevant deviations between the four isolate groups were detected regarding various nutrients beyond the selection, which are also described below.

#### 3.2.3. Relevant Metabolic Varieties between the Four MRSA Groups

##### 3.2.3.1. Metabolic Differences between the Dominant and Rare Lineage within a CC

Different colonization and infection sites within a pathogen’s host require unique metabolic pathways due to the varying availability of nutrients. For example, *S. aureus* would likely have access to lactate, urea and amino acids, but not carbohydrates at skin surfaces, and to serum glucose at deep tissue sites [65]. Moreover, several studies have shown that the metabolic state of *S. aureus* has an impact on the activity of major virulence factor regulators, proving the linkage of *S. aureus*’ metabolism to its overall pathogenesis [65]. Therefore, it seemed likely that the dominant LA-MRSA CC9 from China and CC398 from Germany might have an advantage in host colonization due to certain metabolic properties in relation to the non-successful pig colonizing isolates. 

**Figure 3:**
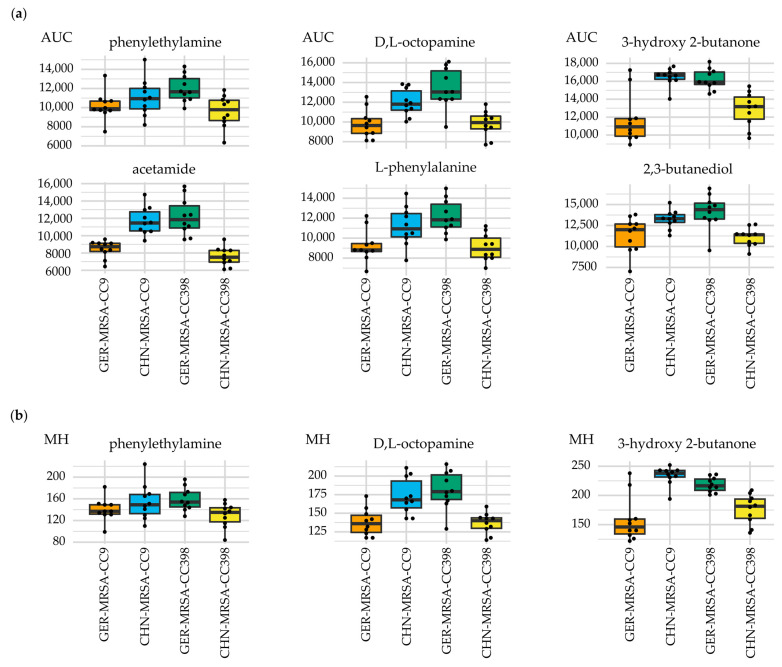
Relevant metabolic differences between the dominant and rare lineage within a CC regarding the usage of the substances given above the individual diagrams. The box plots display varying (**a**) area under the curve (AUC) values and (**b**) maximum height (MH) values of the four isolate groups.

Lower AUC values indicated a less successful substrate metabolization by the rare lineages compared with the respective dominant clone within the same CC for the carbon sources phenylethylamine, D,L-octopamine and 3-hydroxy 2-butanone (Figure 3a). More precisely, the combined sPLS-DA revealed that the associated respiration curves of the dominant clones displayed greater MH values (Figure 3b). In addition, beyond the sPLS-DA selections, lower AUC values indicated a less successful substrate metabolization by the rare lineages compared with the respective dominant clone within the same CC regarding the carbon sources acetamide, L-phenylalanine and 2,3-butanediol (Figure 3a).

In contrast to initial expectations, only a few relevant metabolic differences were found between the dominant and rare lineages within a CC. Thus, the dominant lineages might have only single advantages in environments with limited nutrient availability. However, due to *S. aureus*’ versatility in nutrient utilization and since several substrates can be used alternatively in the same essential metabolic pathways, we speculate that these variations did not play a role in the emergence of two different epidemic LA-MRSA clones in swine. Advantages such as an enhanced biofilm forming ability and an increased tolerance to desiccation, which we revealed in a previous study [23], more likely allow the dominant clones to outcompete the non-successful lineages in extreme environments.

##### 3.2.3.2. Distinctive Metabolic Features of CHN-MRSA-CC9

**Figure 4:**
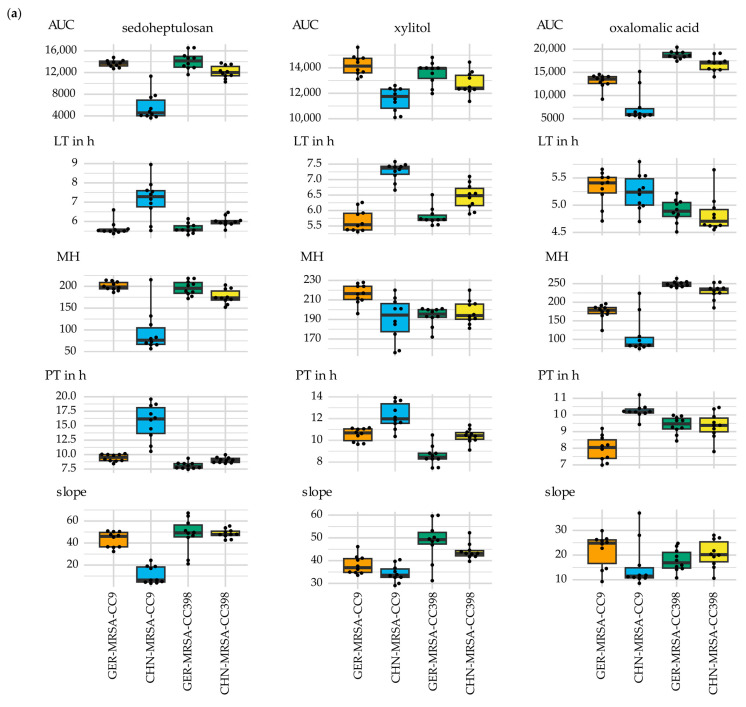

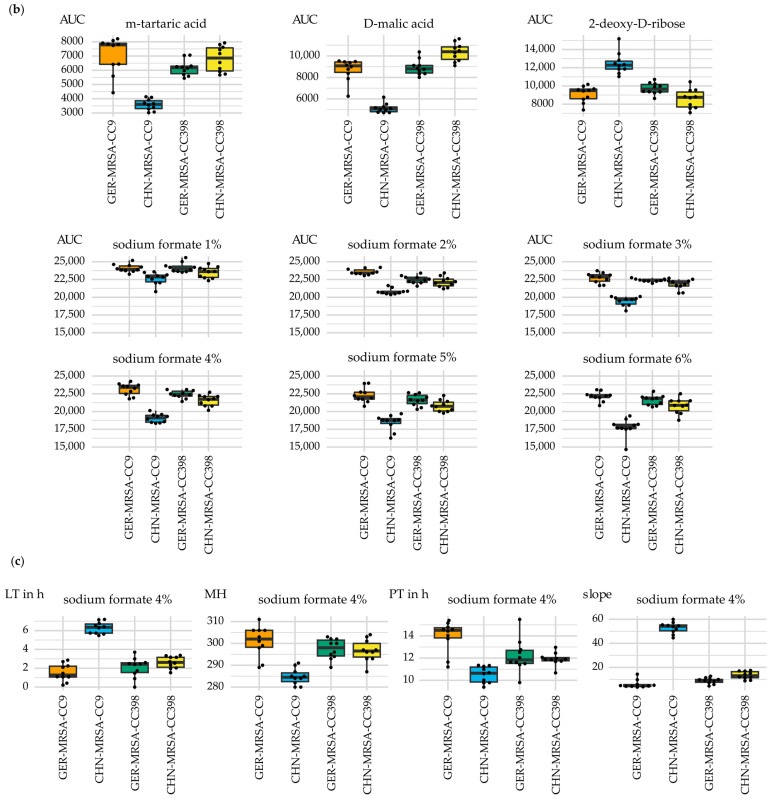
Major differences in the metabolic properties of the CHN-MRSA-CC9 compared with the other three groups regarding the substances or conditions given above the individual charts. The box plots display the varying (**a**) area under the curve (AUC), lag time (LT), maximum height (MH), plateau time (PT) and slope values, (**b**) AUC values and (**c**) LT, MH, PT and slope values of the four isolate groups. LT and PT are given in hours and sodium formate 4% in (**c**) is representative for sodium formate 1% to 6%.

The CHN-MRSA-CC9 showed varying metabolic capacities regarding several substrates compared with the other three groups. Major differences were detected for the carbon sources sedoheptulosan, xylitol and oxalomalic acid. Here, lower AUC values pointed towards a reduced usage in relation to the other groups. According to the combined sPLS-DA, higher PT and lower slope values were recognized for all three substrates. Moreover, an extended LT was observed in the metabolization of sedoheptulosan and xylitol and lower MH values were identified for sedoheptulosan as well as oxalomalic acid (Figure 4a). Sedoheptulosan was also included in the AUC sPLS-DA selection. Regarding the usage of oxalomalic acid, evaluation of the respiration curves provided additional information. Although Figure 4a indicated that the utilization of this nutrient by the CHN-MRSA-CC9 was the lowest, one isolate of the group could use it better than the others (see Section 3.2.3.6). The GER-MRSA-CC9 also seemed to have more difficulties using the substance compared to the MRSA-CC398. Therefore, the differences between the four groups considering oxalomalic acid could also have been discussed in Section 3.2.3.3. Interesting changes in the further progression of the respiration curves were only evaluable considering those resulting from an incubation period of 48 h (see Section 3.2.3.6).

Furthermore, relevant differences were detected beyond the sPLS-DA selections for the carbon sources m-tartaric acid and D-malic acid. Again, lower AUC values indicated a reduced usage in relation to the other groups (Figure 4b). In contrast, respiration curves and AUC values suggested a comparatively increased metabolization of the substrate 2-deoxy-d-ribose (Figure 4b). The AUC values also pointed towards a distinctly increased metabolization of the substrate 3-0-β-D-galactopyranosyl-D-arabinose by the CHN-MRSA-CC9. Evaluation of the respective respiration curves revealed that only these isolates were able to initiate cellular respiration within an incubation period of 24 h (see Section 3.2.3.6). However, the observation of single non-CHN-MRSA-CC9 isolates also being able to metabolize the substance was only detectable over a period of 48 h (see Section 3.2.3.6).

**Figure 5:**
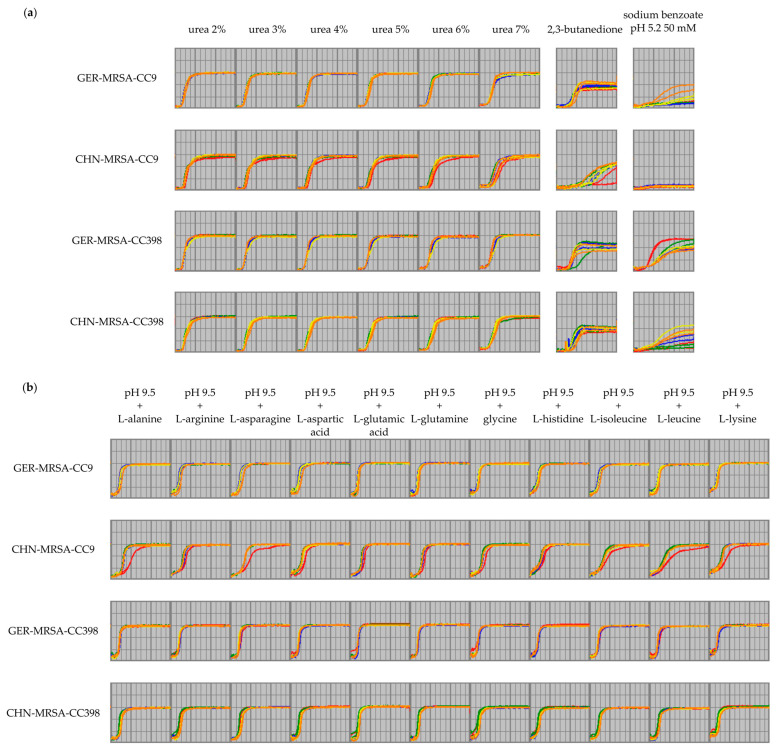
Curves displaying cellular respiration of the four isolate groups over an incubation period of 24 h including two test runs. The isolates within each group are indicated in different colors. (**a**) The CHN-MRSA-CC9 show a delayed onset of metabolization in the presence of urea 2% to 7% as well as 2,3-butanedione and no metabolic activity in the presence of sodium benzoate 50 mM at pH 5.2. (**b**) The CHN-MRSA-CC9 show a delayed onset of cellular respiration considering the usage of different amino acids at a pH value of 9.5.

The reduced metabolic capacity and the delay of metabolism resulting from rising concentrations of sodium formate as mentioned in Section 3.2.1 was recognized for all four groups, but was most distinct for the CHN-MRSA-CC9. Here, the respiration curves displayed lower AUC values, but also an extended LT, lower MH as well as PT values and a greater slope as shown by the combined sPLS-DA (Figure 4b,c). The AUC sPLS-DA selection also suggested sodium formate at concentrations of 3%, 4% and 6% as relevant. As mentioned above, rising concentrations of urea also led to a delay of bacterial respiration, which was notably more distinct for the CHN-MRSA-CC9 compared to the other groups (Figure 5a). Sodium benzoate 50 mM at a pH of 5.2 was also included in the AUC sPLS-DA selection. As discussed in Section 3.2.1, not all isolates could maintain metabolism under these conditions. While lower respiration curves and the delayed onset of metabolic activity displayed in Figure 5a indicated that isolates belonging to all groups struggled using this substance, no metabolic activity was detected for the CHN-MRSA-CC9. Thus, the CHN-MRSA-CC9 showed an increased sensitivity to rising concentrations of sodium benzoate in relation to the other groups (see Section 3.2.1).

In addition, the respiration curves of the CHN-MRSA-CC9 suggested the requirement for a longer adaptation time to the surrounding conditions before onset of nutrient utilization. Not all findings can be shown here, but the observation was particularly evident considering the usage of 2,3-butanedione (Figure 5a) as well as of amino acids and derivatives thereof at basic pH values. The examples given in Figure 5b are representative for all amino acids and derivatives at pH 9.5 and several of them were also emphasized by the AUC sPLS-DA (see Section 3.2.2.1). The availability of amino acids, which can be catabolized to produce the essential biosynthetic intermediates, is of importance for *S*. *aureus*, as the bacterium is frequently described as an amino acid auxotroph [61]. However, *S*. *aureus* often has the ability to revert to a prototrophic state, which is important for the adaptation to host/environmental niches where nutrients are limited [61]. Moreover, Halsey et al. have shown that certain amino acids serve as central carbon sources for *S*. *aureus* critical for growth in medium lacking glucose [66]. This supports a model describing the ability to metabolize secondary carbon sources, such as amino acids, as essential for survival in niches where preferred nutrients, such as glucose, are limited, as for example within a staphylococcal abscess [66]. Thus, the CHN-MRSA-CC9 might be inferior in niche adaptation due to their delayed amino acid catabolism.

As expected, the cgMLST revealed a closer relationship between the isolates within the two CCs regardless of their geographical origin. Nevertheless, the CHN-MRSA-CC9 showed major metabolic differences to the other three groups. The greater similarities of the CHN-MRSA-CC398, GER-MRSA-CC398 and GER-MRSA-CC9 are also displayed in Figure 2. In a previous study, we observed that the MRSA-CC9 from China and Germany possibly belong to two independent evolutionary lineages, whilst the CHN-MRSA-CC398 and the GER-MRSA-CC398 originated from the same ancestor [23]. Moreover, we speculated that the MRSA-CC398 might have been introduced into China through trading activities [23]. Another recent study also revealed a close evolutionary relationship between Chinese and European or Australian LA-MRSA ST398 [16]. The greater spatial distance of the CHN-MRSA-CC9 to the other three lineages during evolution of epidemic clones in swine might explain the more distinct metabolic profile of the MRSA-CC9 from China. The carriage of a larger number of antimicrobial resistance genes has possibly led to a higher fitness cost in the CHN-MRSA-CC9 than in other isolates [67], explaining the reduced metabolic capabilities regarding several substances and the throughout extended adaptation times before onset of cellular respiration. Increased metabolization of 2-deoxy-d-ribose and 3-0-β-D-galactopyranosyl-D-arabinose by the CHN-MRSA-CC9 in relation to the other groups may, however, represent a slight advantage for these isolates, but might as well result in notable disadvantages in case of harmful metabolite formation during metabolic processes. 

In the previous study, we also suggested that the current epidemic MRSA-CC9 clone in China may be gradually replaced by the MRSA-CC398 lineage due to several advantageous features that allow MRSA-CC398 to persist in the pig host or hostile environment [23]. These included easier integration of foreign antimicrobial resistance genes, improved fitness and adaptation to varying environmental conditions, enhanced virulence in infection models, increased pathogenicity due to the *hysA*^νSaβ^ gene, better adaptation to acidic and hyperosmotic environments and higher competitiveness compared to MRSA-CC9 [23]. The small disadvantages of the CHN-MRSA-CC9 in metabolic properties described in this study may support the replacement of the epidemic Chinese clone by MRSA-CC398 after further dissemination of isolates through livestock trade or human occupational exposure. The delayed onset of cellular respiration of the CHN-MRSA-CC9, combined with the decreased usage of many nutrients as well as possible disadvantages resulting from increased metabolization rates of some substrates might be sufficient for MRSA-CC398 to become established once extensively colonizing the pig host. Despite the metabolic robustness typical for *S*. *aureus* [61], the CHN-MRSA-CC9 may be less adaptable to environmental challenges than the MRSA-CC398 clone. The increasing reports of swine-associated MRSA-CC398 isolates from China support the hypothesis of the ongoing MRSA-CC9 replacement process [16,17]. It is important to note that in the future MRSA-CC398 may pose a greater public health threat than MRSA-CC9 due to their enhanced pathogenicity [23]. For example, results from Cui et al. recently suggested that ST398 was a frequent source of MRSA and MSSA infections in the Qinghai province [16]. Li et al. even implied a potential MRSA-ST398 transmission among different countries [17]. They reported an MRSA ST398 isolate obtained from an infected patient in Europe that differed by only 31 SNPs from the airborne dust-associated Chinese isolate in their study [17]. This highlights the need for comprehensive worldwide LA-MRSA monitoring.

##### 3.2.3.3. Metabolic Diversity Based on the Assignment to a CC

**Figure 6:**
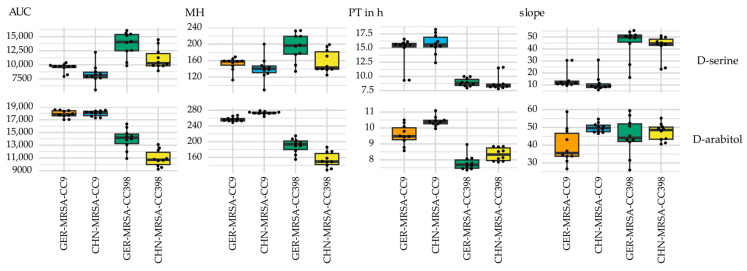
Box plots selected by combined sPLS-DA showing varying area under the curve (AUC), maximum height (MH), plateau time (PT) and slope values of the four isolate groups regarding the metabolization of D-serine and D-arabitol, indicating a CC-dependent nutrient usage. The PT is given in hours.

Respiration curves resulting from the metabolization of D-serine and D-arabitol indicated a CC-dependent usage of these nutrients in accordance with the combined sPLS-DA variable selection. Considering D-serine, the MRSA-CC9 isolates displayed lower AUC, MH and slope values, but an extended PT in relation to the MRSA-CC398 isolates (Figure 6). In contrast, higher AUC, MH and PT values indicated an increased metabolic activity of the MRSA-CC9 compared to the MRSA-CC398 isolates regarding D-arabitol (Figure 6). The substrate was also included in the AUC sPLS-DA selection.

In a previous study [23] we identified several advantages of MRSA-CC398 isolates towards MRSA-CC9 as they revealed more diverse genome structures, higher tolerance to acids and high osmotic pressure and greater competitive fitness in co-culture experiments [23]. Moreover, a novel *hysA*^νSaβ^ gene causing enhanced pathogenicity, which was present in CC398 isolates but absent in MRSA-CC9, might explain why MRSA-CC398 has been reported to be more likely to cause infections [6,23,68]. For this reason, we initially expected to detect multiple differences in the metabolic properties of MRSA-CC9 and MRSA-CC398. However, only single metabolic differences due to CC affiliation were recognized in this study overall.

##### 3.2.3.4. Metabolic Diversity Attributed to the Country of Origin

**Figure 7:**
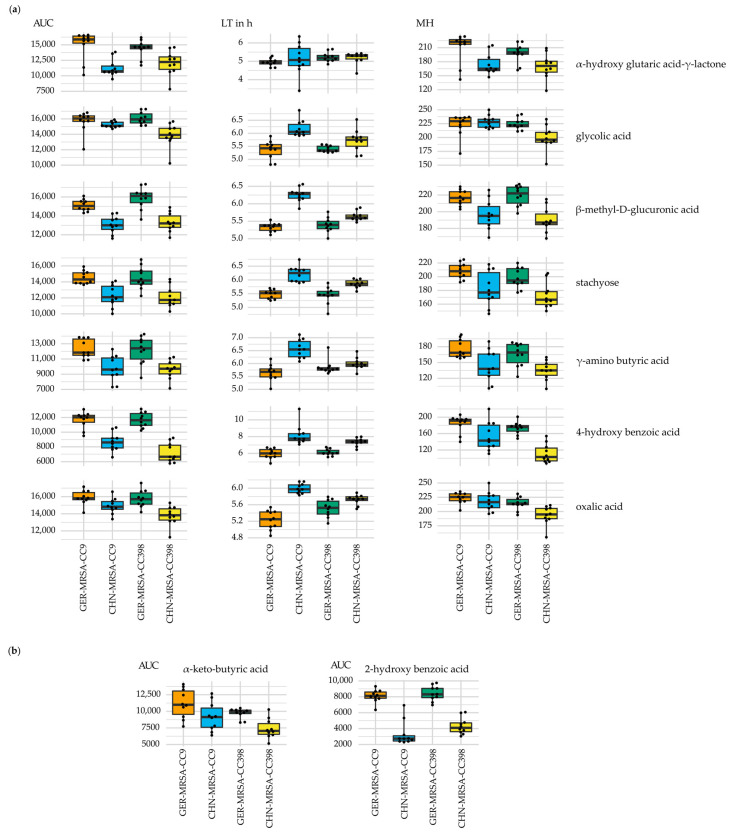
Metabolic differences associated with the isolates’ origin. The box plots illustrate varying (**a**) area under the curve (AUC), lag time (LT) and maximum height (MH) values of the four isolate groups regarding the usage of the nutrients given on the right hand side of the individual diagrams with the LT given in hours and (**b**) AUC values of the four isolate groups regarding the usage of α-keto-butyric acid and 2-hydroxy benzoic acid.

Considering the carbon sources α-hydroxy glutaric acid-γ-lactone, glycolic acid, β-methyl-D-glucuronic acid, stachyose, γ-amino butyric acid, 4-hydroxy benzoic acid and oxalic acid in the combined sPLS-DA selection, differences in metabolic properties seemed to be associated with the isolates’ origin. Throughout, the Chinese MRSA showed lower AUC and MH values as well as an extended LT in comparison with the German isolates, indicating a decreased nutrient usage (Figure 7a). Moreover, as shown by lower AUC values beyond the sPLS-DA selections, the CHN-MRSA-CC9 and CHN-MRSA-CC398 appeared to be less successful in metabolizing α-keto-butyric acid and especially 2-hydroxy benzoic acid compared to the German MRSA (Figure 7b). These findings may suggest an adaptation of the MRSA isolates to the unique character of the Chinese and German pig farm environments.

##### 3.2.3.5. Other Metabolic Differences between the Groups

Some variations in the metabolic activity between the four groups CHN-MRSA-CC9, CHN-MRSA-CC398, GER-MRSA-CC9 and GER-MRSA-CC398 were individual observations and, thus, not discussed in detail. An example is given in Figure S1. According to the combined sPLS-DA, higher AUC and MH values and a shortened LT indicated a more successful usage of glycine by the GER-MRSA-CC398 compared to the other three groups.

In addition, variable results were detected for certain nutrients within each of the four groups and, therefore, not categorized further. Here, the metabolization of nutrients seemed to be more isolate-dependent. Selected examples displaying cellular respiration in the presence of dulcitol, tricarballylic acid, glycogen, a-methyl-D-glucoside, sodium lactate 3% or sodium nitrite 40 mM are shown in Figure S2. Sodium lactate 3% is representative for sodium lactate 2% to 12% and sodium nitrite 40 mM is representative for sodium nitrite 10 mM to 100 mM.

##### 3.2.3.6. Metabolic Variations Detectable Only after a 48 h Incubation Period

**Figure 8:**
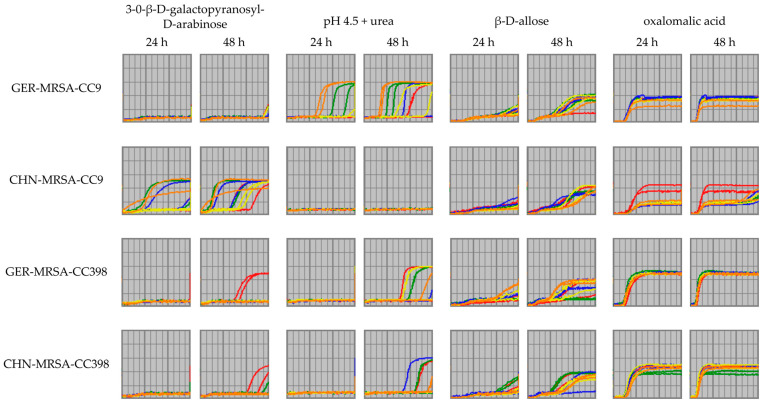
Curves displaying cellular respiration of the four isolate groups in the presence of 3-0-β-D-galactopyranosyl-D-arabinose, pH 4.5 + urea, β-D-allose and oxalomalic acid over an incubation period of 24 h and 48 h including two test runs. The isolates within each group are indicated in different colors. Here, interesting changes in curve progression are only detectable by investigating the 48 h respiration curves additionally.

Some disparities in the isolates’ metabolic activities were only detectable after an incubation period of 48 h. For the substance 3-0-β-D-galactopyranosyl-D-arabinose, this has already been mentioned in Section 3.2.3.2 (Figure 8). In addition, the metabolization of urea at a pH of 4.5 would have been interpreted differently, if only the 24 h interval would have been considered. The 24 h respiration curves suggested that only two GER-MRSA-CC9 were able to initiate metabolic activity. In contrast, the curves obtained after 48 h of incubation revealed that more isolates from several groups were able to utilize urea at a pH of 4.5 after an adaptation period. However, the CHN-MRSA-CC9 were unable to metabolize this substrate under these conditions (Figure 8). 

The 48 h values also enabled the identification of changes in curve progression and thus of differences between the groups that otherwise would not have been recognized (Figure 8). According to the 24 h respiration curves, all four groups seemed to have difficulties in using the nutrient β-D-allose, but only the 48 h curves provided information on the further course. It was demonstrated for all groups that substrate utilization was possible after a period of adaptation. However, differing durations of adjustment indicated that the ability was not evenly distributed within the four groups. In general, the CHN-MRSA-CC9 required the longest adaptation time (Figure 8). The reduced usage of oxalomalic acid by the CHN-MRSA-CC9 has already been mentioned in Section 3.2.3.2. The CHN-MRSA-CC9 displayed a slightly delayed onset of cellular respiration and all isolates except one showed only very low levels of metabolic activity in relation to the other isolates. The 24 h respiration curves did not suggest changes in course progression at a later point in time. However, the 48 h respiration curves of four CHN-MRSA-CC9 showed an increase towards the end of the incubation period almost up to the level of the other MRSA-CC9 isolates (Figure 8). 

Finally, the examples referring to urea at pH 4.5, β-D-allose and oxalomalic acid also supported the conclusion that, compared to the other three groups, the CHN-MRSA-CC9 required a longer period of adaptation before the onset of cellular respiration and/or are not able to metabolize several substances at all or only to a lesser extent.

### 3.3. Potential Limitations of the Study

The aim of this study was to conduct a comparative evaluation of the metabolic properties of four MRSA groups with only five isolates per group and thus, 20 isolates in total. This is a relatively small sample size and might limit the transferability of the results for the represented groups. Considering the MRSA-CC398 from China, the isolates investigated were the only ones available to us during the study period. Due to limited resources, we decided to assemble comparable groups. Preliminary experiments confirmed, in general, a uniform metabolic behavior of the isolates within each group. For this reason, it was possible to draw the respective conclusions from the results obtained analyzing this limited isolate collection.

Only four Biolog PM microplate layouts out of ten possible metabolic phenotyping arrays available for bacteria and fungi provided by Biolog Inc. (Hayward, CA, USA) were used for the experiments. The microplate layouts PM1 (carbon utilization), PM2A (carbon utilization), PM9 (osmotic and ionic effects) and PM10 (pH effects) were chosen, because here we expected to observe relevant differences between the four MRSA groups. Cellular stress responses and a high osmotolerance allow *S. aureus* to persist in different environmental settings. In addition, despite its versatility in nutrient usage, *S. aureus* is known for typically metabolizing carbohydrates when they are available and the carbohydrate catabolism has been studied most extensively [61]. However, the application of additional Biolog PM layouts, e.g., phosphorus and sulfur sources, might be of interest for possible future studies. It should be noted that the PM5 (biosynthetic pathways) layout is not appropriate for testing staphylococci because of their polyauxotrophy [69].

Another potential limitation might be that no standardized statistical pipeline was available that would allow us to compare Biolog PM assay data not only between several bacterial isolates but also between the four isolate groups. For this reason, the AUC sPLS-DA and the combined sPLS-DA were carried out in an attempt to select the most relevant metabolic variations out of the extensive dataset. However, manual comparison of all respiration curves revealed substrates and/or conditions beyond the preselections as relevant due to notable metabolic variations between the four groups. Therefore, an automated, less work-intensive analysis of the data was not possible, although AUC sPLS-DA and combined sPLS-DA proved to be helpful.

Finally, it is worth noting that bacterial metabolism is a large and complex field. Biochemical processes and the regulatory interactions between them have been partially elucidated, but are still unknown to a large extent [70]. In order to generate insight into metabolic networks, integration of experimental results with published data, as well as accumulated observations and anecdotal tales exchanged among scientists working in this field is required [70]. However, a decline in research focusing on microbial metabolism would appear to make this more difficult [70]. Therefore, a limitation of this study may be that we were not able to discuss all substrates in detail that were mentioned in the context of relevant metabolic differences between the MRSA groups. Despite extensive literature research, which metabolic pathways of *S*. *aureus* involving these substrates play a role in fitness and niche competition in a given clinical or environmental setting is not known. It was also not possible to assign the respective substrates to specific groups, other than carbon sources, that would have allowed general conclusions to be drawn. Thus, further research integrating in silico, in vitro and in vivo approaches is required to better understand microbial metabolism in its details [70].

## 4. Conclusions

Applying Biolog PM technology, we characterized the metabolic profiles of rare as well as widespread porcine LA-MRSA from Germany and China. Overall, differences between the four LA-MRSA groups were rather small. In contrast to initial expectations, only a few metabolic differences were found between the dominant and rare lineages within the same CC. Therefore, unique metabolic profiles most likely did not play a key role in the formation of the dominant clones CHN-MRSA-CC9 and GER-MRSA-CC398, respectively. Surprisingly, the most frequent and substantial metabolic variations were detected between the CHN-MRSA-CC9 and the other three LA-MRSA groups. In addition to reduced or even absent usage of some substrates, and the increased metabolization of 2-deoxy-d-ribose and 3-0-β-D-galactopyranosyl-D-arabinose, especially the delayed onset of cellular respiration was often noticed. The AUC sPLS-DA heat map also suggested metabolic differences of the CHN-MRSA-CC9 compared to the other groups. These comparatively unfavorable metabolic properties might promote the suspected already ongoing gradual replacement of the current epidemic MRSA-CC9 clone in China by the superior MRSA-CC398 clone. Thus, the risk for public health posed by LA-MRSA originating from swine might rise alarmingly in the future due to the enhanced pathogenicity of the MRSA-CC398 lineage.

## Figures and Tables

**Table 1:** Characteristics of the representative bacterial isolates of swine origin selected for Biolog PM assays.

ID	Year	MLST	*spa*	*dru*	SCC*mec*	Resistance Phenotype ^1^	Resistance Genotype ^2^
**CHN-MRSA-CC9**							
DL44	2009	ST9	t899	dt12w	XII(9C2)	bla, tet, ery, cli, gen, cip, ffn, tia, tmp	*mecA, blaZ* (2x), *tet*(L), *erm*(C), *lnu*(B), *lsa*(E), *aacA-aphD*, *aadD*, *aadE*, *spw*, *cat(pC221), fexA*, *dfrG* ^3,4^
QDT9	2014	ST9	t899	dt12w	XII(9C2)	bla, tet, ery, cli, gen, cip, ffn, tia, tmp	*mecA*, *blaZ* (2x), *tet*(L), *erm*(C), *lnu*(B), *lsa*(E), *aacA-aphD*, *aadD*, *aadE*, *spw*, *fexA*, *dfrG* ^3,4^
PNB35	2009	ST9	t899	dt12w	XII(9C2)	bla, tet, ery, cli, gen, cip, ffn, tia, tmp	*mecA, blaZ, tet*(L), *lnu*(B), *lsa*(E), *aadD*, *aadE*, *spw*, *fexA*, *dfrG* ^3,4^
SF30	2013	ST9	t899	dt8av	XII(9C2)	bla, tet, ery, cli, gen, cip, ffn, tia, tmp	*mecA*, *blaZ* (2x), *tet*(L), *lnu*(B), *lsa*(E), *aadD*, *aadE*, *spw*, *fexA*, *dfrG* ^3,4^
DY82	2014	ST9	t1939	dt10du	XII(9C2)	bla, tet, ery, cli, gen, cip, ffn, tia, tmp	*mecA*, *blaZ*, *tet*(K), *tet*(L), *erm*(C), *lnu*(B), *lsa*(E)*, aacA-aphD*, *aadD*, *aadE*, *spw*, *str*, *fexA*, *dfrG* ^4^
**CHN-MRSA-CC398**							
GDC1 ^5^	2016	ST398	t034	dt11ax	Vc(5C2&5)	bla, tet, ery, cli, ffn, tia, tmp	*mecA*, *blaZ*, *tet*(K), *tet*(M), *erm*(C), *lnu*(B), *lsa*(E), *spc*, *fexA*, *dfrG*, *cfr*
SHP1 ^5^	2016	ST398	t571	dt9bw	V(5C2)	bla, tet, ery, cli, gen, cip, tia, tmp	*mecA*, *blaZ* (2x), *tet*(L), *tet*(M), *erm*(T), *lnu*(B), *lsa*(E), *aacA-aphD*, *aadD*, *aadE*, *spw*, *fexA*, *dfrG* ^3^
YN471	2017	ST398	t034	dt6j	Vc(5C2&5)	bla, tet, ery, cli, tia, tmp	*mecA*, *blaZ*, *tet*(K), *tet*(M), *erm*(A), *vga*(E), *spc*, *dfrG*
YN523	2017	ST398	t034	dt6j	Vc(5C2&5)	bla, tet, ery, cli, tia, tmp	*mecA*, *blaZ*, *tet*(K), *tet*(M), *erm*(A), *vga*(E), *spc*, *dfrG*
YN502	2017	ST398	t034	dt6j	Vc(5C2&5)	bla, tet, ery, cli, tia, tmp	*mecA*, *blaZ*, *tet*(K), *tet*(M), *erm*(A), *erm*(C), *vga*(E), *spc*, *dfrG*
**GER-MRSA-CC9**							
DG34	2015	ST9	t15199	dt10a	IV(2B)	bla, cip	*mecA*, *blaZ*, *str* ^3,4^
DG36	2015	ST9	t1430	dt10a	IV(2B)	bla, cip	*mecA*, *blaZ*, *str* ^3,4^
DG37	2015	ST9	t1430	dt10a	IV(2B)	bla, cli, cip	*mecA*, *blaZ*, *str*^3,4^
DG39	2015	ST9	t1430	dt10a	IV(2B)	bla, tet, ery, cli, cip, tmp	*mecA*, *blaZ*, *tet*(L), *erm*(B), *aadD*, *dfrK*^3,4^
DG41	2017	ST9	t1430	dt10a	IV(2B)	bla, cli, cip	*mecA*, *blaZ*, *str* ^3,4^
**GER-MRSA-CC398**							
DG4	2008	ST398	t011	dt11a	Vc(5C2&5)	bla, tet	*mecA*, *blaZ*, *tet*(K), *tet*(M), *str*
DG9	2008	ST398	t011	dt11a	Vc(5C2&5)	bla, tet, tmp	*mecA*, *blaZ*, *tet*(K), *tet*(L), *tet*(M), *str*, *dfrK*
DG12	2008	ST398	t011	dt11a	Vc(5C2&5)	bla, tet, ery, cli, gen, tmp	*mecA*, *blaZ*, *tet*(K), *tet*(M), *erm*(C), *str*, *dfrG*
DG17	2008	ST398	t011	dt11a	Vc(5C2&5)	bla, tet, ery, cli, cip	*mecA*, *blaZ*, *tet*(K), *tet*(M), *erm*(C), *str* ^3^
DG29	2004	ST398	t011	dt11a	Vc(5C2&5)	bla, tet, cli, gen	*mecA*, *blaZ*, *tet*(K), *tet*(M), *aacA-aphD*, *str*

^1^ bla, β-lactam antibiotics; tet, tetracycline; ery, erythromycin; cli, clindamycin; gen, gentamicin; cip, ciprofloxacin; ffn, florfenicol; tia, tiamulin; tmp, trimethoprim. Despite the lack of clinical breakpoints approved by the Clinical and Laboratory Standards Institute (CLSI), isolates that showed high minimal inhibitory concentration values for ffn (≥32 mg/L) and tia (≥16 mg/L) were considered as resistant. ^2^ All isolates carried the gene *tet*(38), which may confer resistance to tetracycline when overexpressed [31]. Its presence alone is not associated with tetracycline resistance, because it can be found in nearly every *S*. *aureus* genome including phenotypically susceptible isolates. ^3^ These isolates harbored fluoroquinolone resistance-mediating point mutations in *gyrA* and *grlA* (S84A/S80F—DL44, QDT9, PNB35, SF30; S84L/S80Y—SHP1; S84L/S80F—DG34, DG36, DG37, DG39, DG41, DG17) [32,33]. ^4^ These isolates harbored the quinolone resistance-mediating type A mutation in the *norA* promoter region [34]. ^5^ GDC1 corresponds to GDC6P096P and SHP1 corresponds to SHP6P021P in a previous study [23].

## Data Availability

All data presented in this study are available in the text, figures and tables of the main article and in the supplementary material. Whole-genome sequences of the MRSA isolates included in this study are available at GenBank under the accession numbers JAMYYK000000000, JAMYYJ000000000, JAMYYI000000000, JAMYYH000000000, JAMYYG000000000, JAMYYF000000000, JAMYYE000000000, JAMYYD000000000, JAMYYC000000000, JAMYYB000000000, JAMYYA000000000, JAMYXZ000000000, JAMYXY000000000, JAMYXX000000000, JAMYXW000000000, JAMYXV000000000, JAMYXU000000000, JAMYXT000000000, JAMYXS000000000 and JAMYXR000000000.

## Supplementary Materials

The following supporting information can be downloaded at: https://www.mdpi.com/article/10.3390/microorganisms10112116/s1, Table S1: Distance matrix of the core genome allelic profiles of the 20 representative porcine MRSA from Germany and China including 1749 of 1861 possible target genes by removing 112 columns with missing values from the comparison table created with the SeqSphere+ software, Table S2: Biolog PM microplate wells that showed abiotic “false-positive” reactions under the study’s test conditions, Figure S1: Metabolization of glycine by the four isolate groups, and Figure S2: Selected Biolog PM assay results that were variable within the four isolate groups and, therefore, not categorized further.
