# Supplementary material for: Metabolic Characteristics of Porcine LA-MRSA CC398 and CC9 Isolates from Germany and China via Biolog Phenotype MicroArrayTM
Source: Microorganisms. 2022 Oct 26;10(11):2116. doi: 10.3390/microorganisms10112116 (PMC9693340; doi:10.3390/microorganisms10112116)
Supplement: Supplementary file 1 [file microorganisms-10-02116-s001.zip › microorganisms-1974046-supplementary.pdf]

Table S1. Distance matrix of the core genome allelic profiles of the 20 representative porcine MRSA from Germany and China including 1749 of 1861 possible target genes by removing 112 columns with missing values from the comparison table created with the SeqSphere+ software.

	DL44	QDT9	PNB35	SF30	DY82	GDC1	SHP1	YN471	YN523	YN502	DG34	DG36	DG37	DG39	DG41	DG4	DG9	DG12	DG17	DG29
**DL44**	0	55	47	43	86	1658	1559	1659	1659	1659	195	192	200	191	196	1655	1656	1655	1657	1655
**QDT9**	55	0	68	64	108	1659	1565	1660	1660	1660	212	209	217	208	212	1656	1657	1656	1658	1656
**PNB35**	47	68	0	53	92	1658	1560	1659	1659	1659	196	194	202	193	197	1655	1656	1655	1657	1655
**SF30**	43	64	53	0	92	1658	1560	1659	1659	1659	199	195	203	195	198	1655	1656	1655	1657	1655
**DY82**	86	108	92	92	0	1655	1560	1656	1656	1656	223	219	227	219	222	1653	1654	1653	1655	1653
**GDC1**	1658	1659	1658	1658	1655	0	241	119	114	119	1663	1662	1662	1662	1662	124	127	128	143	122
**SHP1**	1559	1565	1560	1560	1560	241	0	221	218	222	1571	1572	1571	1570	1573	230	234	238	247	230
**YN471**	1659	1660	1659	1659	1656	119	221	0	8	14	1664	1663	1663	1663	1663	106	110	113	125	105
**YN523**	1659	1660	1659	1659	1656	114	218	8	0	12	1664	1663	1663	1663	1663	104	108	111	123	103
**YN502**	1659	1660	1659	1659	1656	119	222	14	12	0	1664	1663	1663	1663	1663	106	110	113	125	105
**DG34**	195	212	196	199	223	1663	1571	1664	1664	1664	0	31	41	25	35	1660	1661	1660	1662	1660
**DG36**	192	209	194	195	219	1662	1572	1663	1663	1663	31	0	34	26	28	1659	1660	1659	1661	1659
**DG37**	200	217	202	203	227	1662	1571	1663	1663	1663	41	34	0	36	31	1659	1660	1659	1661	1659
**DG39**	191	208	193	195	219	1662	1570	1663	1663	1663	25	26	36	0	30	1659	1660	1659	1661	1659
**DG41**	196	212	197	198	222	1662	1573	1663	1663	1663	35	28	31	30	0	1659	1660	1659	1661	1659
**DG4**	1655	1656	1655	1655	1653	124	230	106	104	106	1660	1659	1659	1659	1659	0	16	23	36	15
**DG9**	1656	1657	1656	1656	1654	127	234	110	108	110	1661	1660	1660	1660	1660	16	0	25	37	17
**DG12**	1655	1656	1655	1655	1653	128	238	113	111	113	1660	1659	1659	1659	1659	23	25	0	42	14
**DG17**	1657	1658	1657	1657	1655	143	247	125	123	125	1662	1661	1661	1661	1661	36	37	42	0	35
**DG29**	1655	1656	1655	1655	1653	122	230	105	103	105	1660	1659	1659	1659	1659	15	17	14	35	0

Table S2. Biolog PM microplate wells that showed abiotic "false-positive" reactions under the study's test conditions.

Biolog PM microplate	well	substrate
PM1 MicroPlate™ Carbon Sources	A02	L-arabinose
	A12	D-mannose
	B08	D-xylose
	C04	D-ribose
	H06	L-lyxose
PM2A MicroPlate™ Carbon Sources	B05	D-arabinose
	B09	2-deoxy-D-ribose
	E05	D-glucosamine
	E12	5-keto-D-gluconic acid
	F09	sorbic acid
	H09	dihydroxy acetone
PM9 MicroPlate™ Osmolytes	A06	NaCl 5.5%
	A07	NaCl 6%
	A10 – A12	NaCl 8%, NaCl 9%, NaCl 10%
	B01 – B03	NaCl 6%, NaCl 6% + Betaine, NaCl 6% + N-N dimethyl glycine
	B06	NaCl 6% + MOPS
	B08 – B12	NaCl 6% + choline, NaCl 6% + phosphoryl choline, NaCl 6% + creatine, NaCl 6% + creatinine, NaCl 6 + L-carnitine
	C01	NaCl 6% + KCl
	C02	NaCl 6% + L-proline
	C06	NaCl 6% + glutathione
	C08	NaCl 6% + trehalose
	C10 – C12	NaCl 6% + trimethylamine, NaCl 6% + octopine, NaCl 6% + trigonelline
	D02 – D03	potassium chloride 4%, potassium chloride 5%
	F12	sodium lactate 12%
	G01 – G04	sodium phosphate pH 7 20 mM, sodium phosphate pH 7 50 mM, sodium phosphate pH 7 100 mM, sodium phosphate pH 7 200 mM
	G08	sodium benzoate pH 5.2 200 mM
	G10	ammonium sulfate pH 8 20 mM
	G12	ammonium sulfate pH 8 100 mM
PM10 MicroPlate™ pH	A07 – A12	pH 7, 8, 8.5, 9, 9.5, 10
	D01	pH 4.5 + anthranilic acid
	D05	pH 4.5 + p-amino-benzoic acid
	D09	pH 4.5 + 5-hydroxy tryptophan
	E01 – E12	pH 9.5, pH 9.5 + L-alanine, pH 9.5 + L-arginine, pH 9.5 + L-asparagine, pH 9.5 + L-aspartic acid, pH 9.5 + L-glutamic acid, pH 9.5 + L-glutamine, pH 9.5 + glycine, pH 9.5 + L-histidine, pH 9.5 + L-isoleucine, pH 9.5 + L-leucine, pH 9.5 + L-lysine
	F01 – F12	pH 9.5 + L-methionine, pH 9.5 + L-phenylalanine, pH 9.5 + L-proline, pH 9.5 + L-serine, pH 9.5 + L-threonine, pH 9.5 + L-tryptophan, pH 9.5 + L-tyrosine, pH 9.5 + L-valine, pH 9.5 + hydroxy-L-proline, pH 9.5 + L-ornithine, pH 9.5 + L-homoarginine, pH 9.5 + L-homoserine
	G01 – G12	pH 9.5 + L-anthranilic acid, pH 9.5 + L-norleucine, pH 9.5 + L-norvaline, pH 9.5 + agmatine, pH 9.5 + cadaverine, pH 9.5 + putrescine, pH 9.5 + histamine, pH 9.5 + phenylethylamine, pH 9.5 + tyramine, pH 9.5 + creatine, pH 9.5 + trimethylamine-N-oxide, pH 9.5 + urea
	H01 – H12	X-caprylate, X-α-D-glucoside, X-β-D-glucoside, X-α-D-galactoside, X-β-D-galactoside, X-α-D-glucuronide, X-β-D-glucuronide, X-β-D-glucosaminide, X-β-D-galactosaminide, X-α-D-mannoside, X-PO4, X-SO4

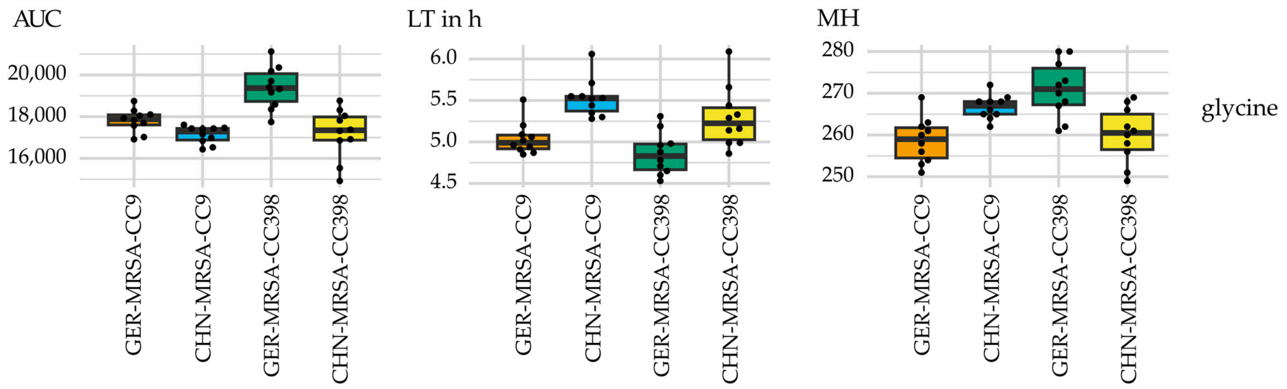

Figure S1. Metabolization of glycine by the four isolate groups. The box plots selected via combined sPLS-DA display area under the curve (AUC), lag time (LT) and maximum height (MH) values. The LT is given in hours. This finding was not discussed further in the main text, because this pattern of varying metabolic activity between the four groups – GER-MRSA-CC398 outcompeting the other groups – was not detected considering other substrates and/or conditions tested.

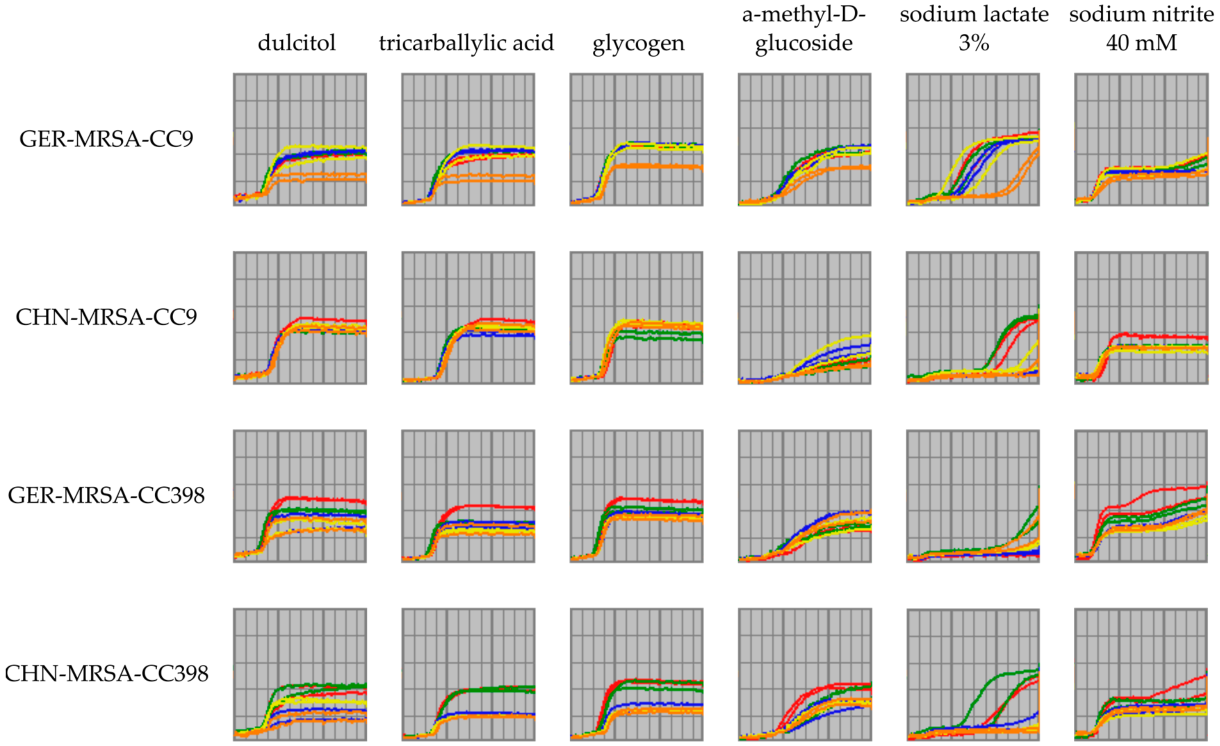

Figure S2. Selected Biolog PM assay results that were variable within the four isolate groups and therefore not categorized further. The curves display cellular respiration over an incubation period of 24 h in the presence of dulcitol, tricarballylic acid, glycogen, a-methyl-D-glucoside, sodium lactate 3%, or sodium nitrite 40 mM. Sodium lactate 3% is representative for sodium lactate 2% to 12% and sodium nitrite 40 mM is representative for sodium nitrite 10 mM to 100 mM. The isolates within each group are indicated in different colors and two test runs are included.
